# Male partner involvement in increasing the uptake of infant antiretroviral prophylaxis/treatment in sub Saharan Africa: A systematic review and meta-analysis

**DOI:** 10.1186/s12889-018-5171-9

**Published:** 2018-02-13

**Authors:** Noah F. Takah, Jeannine A. Atem, Leopold N. Aminde, Moffat Malisheni, Grant Murewenhema

**Affiliations:** 1 Department of Maternal and Child Health, Limbe Regional Hospital, Ministry of Public Health, Limbe Health District, Limbe, South West Region Cameroon; 2 Department of Clinical Research, London School of Hygiene and Tropical Medicine, Harare, UK; 3 Faculty of Health Sciences, University of Buea, Buea, Cameroon; 4 School of Public Health, University of Queensland, Brisbane, Australia; 5 Ministry of Health, Lusaka, Zambia; 6 Ministry of Health Zimbabwe, Limbe, Cameroon

## Abstract

**Background:**

Infant antiretroviral prophylaxis plays an important role towards ensuring the reduction of HIV transmission from mother to child in the postpartum period. However in sub Saharan Africa (SSA), the low level of involvement of male partners may hinder the uptake of such services by HIV positive mothers. We conducted a systematic review and meta-analysis to determine the impact of male partner involvement approaches on the uptake of infant antiretroviral prophylaxis in SSA.

**Methods:**

In this systematic review and meta-analysis, Ovid Medline, Embase, PsycINFO, Cochrane library, ClinicalTrials.gov, Web of Science and Current Controlled Trials were searched from 1st December 2015 up until 30th March 2016. Only studies carried out in SSA that reported an approach used in involving male partners and the impact on the uptake of infant antiretroviral prophylaxis irrespective of the Language and date of publication were included. Odds ratios were extracted or calculated from studies and combined in a meta-analysis using the statistical package Stata version 11.0. Forest plots were generated using the random effect model.

**Results:**

From an initial 2316 non-duplicate articles, 09 articles were included in the systematic review and meta-analysis. The pooled unadjusted odds ratio was 2.09(95% CI: 1.31 to 3.36) while the unadjusted odds ratios for enhanced psychosocial interventions (02 studies pooled), complex community interventions (02 studies pooled), verbal encouragement (02 studies pooled) and invitation letters(03 pooled studies) were 3.48(95% CI: 1.42 to 8.53), 1.85(95%CI: 0.85 to 4.03), 2.37(95%CI: 1.22 to 4.61) and 1.81(95%CI: 0.64 to 5.14) respectively. I squared was 89.5%, *p* < 0.001 and the heterogeneity was not explained by any of the variables in meta-regression.

**Conclusion:**

There was stronger evidence for enhanced psychosocial intervention and verbal encouragement in increasing the uptake of infant prophylaxis. The high heterogeneity suggests more studies are needed to draw a definite inference from the meta-analysis. More studies with larger sample sizes that are conducted using similar methods are needed in the future.

**Trial registration:**

Prospero registration number: 42016032673.

## Background

Despite immense efforts directed towards ensuring the elimination of mother to child transmission of HIV, there are still some 2.6 million children below 15 years living with HIV with 91% of the children in sub Saharan Africa (SSA) [1, 2]. Even though the Joint United Nations Programme on HIV/AIDS(UNAIDS) report of 2016 has shown a propitious reduction in the rate of new HIV infections in children, the number of children infected with HIV still remains unacceptably high at 150,000 cases per year [3]. It has been suggested that factors such as: limited resources available to fund HIV programmes; limited access to HIV prevention services; and poorly organized health systems and socio-cultural barriers which have prevented the effective participation of communities in HIV prevention activities may account for the high HIV burden among children in SSA [4].

There are four main aspects of the prevention of mother to child transmission (PMTCT) of HIV cascade that have been outlined by the World Health Organization (WHO) which can be divided into interventions before pregnancy in women of reproductive age, prenatal (antenatal) interventions, intrapartum(perinatal) interventions and postnatal interventions [5]. The WHO guidelines recommends 4–6 weeks of nevirapine or zidovudine as postnatal prophylaxis, with possible extension to 12 weeks for high-risk breastfed infants [6]. Infant antiretroviral prophylaxis plays a crucial role in the prevention of mother to child transmission of HIV [7, 8]. If maternal adherence to antiretroviral prophylaxis of their infants is high, the risk of transmission of vertical transmission of HIV reduces to less than 5% [9]. However, in the complex sociocultural context in SSA, maternal adherence to antiretroviral prophylaxis and the uptake of other PMTCT services are strongly influenced by the involvement of their male partners [10].

Studies in SSA have shown that the non-involvement of male partners instills fear in their pregnant partners and may deter them from taking up services such as HIV testing and antiretroviral prophylaxis [11, 12]. There is evidence to suggest that the involvement of male partners in PMTCT (Prevention of Mother-To Child Transmission) of HIV does not only reduce the risk of pregnant women acquiring HIV but also improves on the uptake of interventions to prevent vertical transmission of HIV [10, 13, 14]. This improvement can be due to better communication and mutual respect between the couple as a result of male partner involvement [15]. Despite this pivotal role played by male partners, evidence currently suggests that their level of involvement is currently low in sub Saharan African settings [16, 17]. The root causes of this low level of male partner involvement are multifaceted in origin but the study by Skovdal et al. suggests that the hegemonic notions of masculinity among men which inherently results in acts that suggest they are stronger, disease free, sexually and reproductive active and resilient, may be at the epicentre of their refusal to participate in PMTCT services [18]. Therefore, interventions to improve on the involvement of male partners in view of ultimately improving on the uptake of infant prophylaxis by HIV seropositive mothers are currently needed.

The evidence that is needed to guide research and policy in the domain of male involvement on the uptake of infant antiretroviral prophylaxis has been contrasting. According to a systematic review and meta-analysis by Brusamento et al. in 2010, male partner involvement had a negative impact on the uptake of PMTCT services by women [19]. The methodology was robust with a comprehensive search strategy and quality assessment of articles but the authors ended up with only a single study. However, with mounting evidence in favour of the positive impact of male partner involvement on PMTCT that were published after the Brusamento et al. study [10, 13, 20–23], it was necessary to conduct an up to date systematic review with meta-analysis to determine the approaches that have been used in improving on male partner involvement in PMTCT and the impact they have had on the uptake of infant antiretroviral prophylaxis by HIV positive mothers in SSA. This study was therefore conducted to determine the interventions/approaches used in improving on male partner involvement in a PMTCT of HIV such as infant antiretroviral prophylaxis in SSA; and to determine the impact of the approaches used on the uptake of infant antiretroviral prophylaxis by HIV positive mothers in SSA.

## Method

### Protocol and registration

This systematic review with meta-analysis was conducted in accordance with the PRISMA (Preferred Reporting Items for Systematic review and Meta-Analysis) statement of 2015. The protocol for this systematic review was registered in the international prospective register of systematic reviews (PROSPERO). The registration number is CRD42016032673. The protocol was published in the British Medical Journal (BMJ) Open [24]. In the protocol we envisaged outcomes such as infant antiretroviral prophylaxis uptake, maternal ART uptake, safe infant feeding options, condom use and family planning. However, we decided to report infant antiretroviral prophylaxis uptake due to the word limits for a single manuscript.

### Eligibility criteria

This review considered studies that were conducted in SSA. Studies out of the SSA were excluded. No restriction was placed on the setting of the study and the language of study. Randomized controlled trials, prospective and retrospective cohort studies, and serial cross sectional studies were included. The studies were included if they provided data on the impact of male partner involvement on infant antiretroviral prophylaxis. One time cross-sectional studies and case-control studies were excluded because they did not present any evidence of the impact of male partner involvement. The participants were HIV positive mothers and their infants.

### Information sources and search strategy

A literature search was carried out from 1st December 2015 up until 31st March 2016. A search strategy was developed by the principal investigator (NFT) with inputs from JAA and LNA using evidence from a US Centre for Disease Control and Prevention study on how to carry out a detailed systematic search in HIV prevention [25]. Six main databases were searched: Ovid Medline, Ovid Embase, Ovid Health and Psychosocial Instruments (HPSI), Psychological Information (PsycINFO), Web of Science and Cochrane library. Current Controlled Trials and ClinicalTrials.gov were searched for ongoing and newly completed trials. A detailed search strategy is shown in Table 1.

**Table 1:** Search Strategy. Embase, Medline and HPSI search strategy

Database: Ovid MEDLINE(R) without Revisions < 1996 to Week 4 March 2016>, Embase < 1996 to week 4 March 2016>, Health and Psychosocial Instruments < 1985 to Week 4 March 2016>
Search Strategy:
1 *HIV/ (31795)
2 *HIV infection/ (236817)
3 human immunodeficiency virus.mp. [mp = ti, ab, ot, nm, hw, kf, px, rx, ui, tn, dm, mf, dv, kw, ac, de, md, sd, so] (349081)
4 human immuno-deficiency virus.mp. [mp = ti, ab, ot, nm, hw, kf, px, rx, ui, an, tn, dm, mf, dv, kw, ac, sh, de, md, ip, vo, pg, sd, jn, pb, yr., ar, bs, bt, cf., dp, ja, pa, so] (344)
5 human immune-deficiency virus.mp. [mp = ti, ab, ot, nm, hw, kf, px, rx, ui, an, tn, dm, mf, dv, kw, ac, sh, de, md, ip, vo, pg, sd, jn, pb, yr., ar, bs, bt, cf., dp, ja, pa, so] (1157)
6 human immunedeficiency virus.mp. [mp = ti, ab, ot, nm, hw, kf, px, rx, ui, an, tn, dm, mf, dv, kw, ac, sh, de, md, ip, vo, pg, sd, jn, pb, yr., ar, bs, bt, cf., dp, ja, pa, so] (31)
7 (human immun* and deficiency virus).mp. [mp = ti, ab, ot, nm, hw, kf, px, rx, ui, an, tn, dm, mf, dv, kw, ac, sh, de, md, ip, vo, pg, sd, jn, pb, yr., ar, bs, bt, cf., dp, ja, pa, so] (856)
8 *AIDS/pc (7243)
9 acquired immune-deficiency syndrome.mp. [mp = ti, ab, ot, nm, hw, kf, px, rx, ui, an, tn, dm, mf, dv, kw, ac, sh, de, md, ip, vo, pg, sd, jn, pb, yr., ar, bs, bt, cf., dp, ja, pa, so] (74090)
10 acquired immunedeficiency syndrome.mp. [mp = ti, ab, ot, nm, hw, kf, px, rx, ui, an, tn, dm, mf, dv, kw, ac, sh, de, md, ip, vo, pg, sd, jn, pb, yr., ar, bs, bt, cf., dp, ja, pa, so] (20)
11 acquired immunedeficiency syndrome.mp. [mp = ti, ab, ot, nm, hw, kf, px, rx, ui, an, tn, dm, mf, dv, kw, ac, sh, de, md, ip, vo, pg, sd, jn, pb, yr., ar, bs, bt, cf., dp, ja, pa, so] (20)
12 (acquired immune* and deficiency syndrome).mp. [mp = ti, ab, ot, nm, hw, kf, px, rx, ui, an, tn, dm, mf, dv, kw, ac, sh, de, md, ip, vo, pg, sd, jn, pb, yr., ar, bs, bt, cf., dp, ja, pa, so] (72961)
13 vertical transmission.mp. [mp = ti, ab, ot, nm, hw, kf, px, rx, ui, an, tn, dm, mf, dv, kw, ac, sh, de, md, ip, vo, pg, sd, jn, pb, yr., ar, bs, bt, cf., dp, ja, pa, so] (15315)
14 vertical infectious disease transmission.mp. [mp = ti, ab, ot, nm, hw, kf, px, rx, ui, an, tn, dm, mf, dv, kw, ac, sh, de, md, ip, vo, pg, sd, jn, pb, yr., ar, bs, bt, cf., dp, ja, pa, so] (8)
15 mother-to-child transmission.mp. [mp = ti, ab, ot, nm, hw, kf, px, rx, ui, an, tn, dm, mf, dv, kw, ac, sh, de, md, ip, vo, pg, sd, jn, pb, yr., ar, bs, bt, cf., dp, ja, pa, so] (7237)
16 Parent-to-child transmission.mp. [mp = ti, ab, ot, nm, hw, kf, px, rx, ui, an, tn, dm, mf, dv, kw, ac, sh, de, md, ip, vo, pg, sd, jn, pb, yr., ar, bs, bt, cf., dp, ja, pa, so] (207)
17 Maternal-to-child transmission.mp. [mp = ti, ab, ot, nm, hw, kf, px, rx, ui, an, tn, dm, mf, dv, kw, ac, sh, de, md, ip, vo, pg, sd, jn, pb, yr., ar, bs, bt, cf., dp, ja, pa, so] (169)
18 maternal-fetal infection transmission.mp. [mp = ti, ab, ot, nm, hw, kf, px, rx, ui, an, tn, dm, mf, dv, kw, ac, sh, de, md, ip, vo, pg, sd, jn, pb, yr., ar, bs, bt, cf., dp, ja, pa, so] (2)
19 MTCT.mp. [mp = ti, ab, ot, nm, hw, kf, px, rx, ui, an, tn, dm, mf, dv, kw, ac, sh, de, md, ip, vo, pg, sd, jn, pb, yr., ar, bs, bt, cf., dp, ja, pa, so] (1607)
20 PMTCT.mp. [mp = ti, ab, ot, nm, hw, kf, px, rx, ui, an, tn, dm, mf, dv, kw, ac, sh, de, md, ip, vo, pg, sd, jn, pb, yr., ar, bs, bt, cf., dp, ja, pa, so] (2168)
21 pPTCT.mp. [mp = ti, ab, ot, nm, hw, kf, px, rx, ui, an, tn, dm, mf, dv, kw, ac, sh, de, md, ip, vo, pg, sd, jn, pb, yr., ar, bs, bt, cf., dp, ja, pa, so] (78)
22 male partner*.mp. [mp = ti, ab, ot, nm, hw, kf, px, rx, ui, an, tn, dm, mf, dv, kw, ac, sh, de, md, ip, vo, pg, sd, jn, pb, yr., ar, bs, bt, cf., dp, ja, pa, so] (6310)
23 spouse*.mp. [mp = ti, ab, ot, nm, hw, kf, px, rx, ui, an, tn, dm, mf, dv, kw, ac, sh, de, md, ip, vo, pg, sd, jn, pb, yr., ar, bs, bt, cf., dp, ja, pa, so] (39866)
24 husband*.mp. [mp = ti, ab, ot, nm, hw, kf, px, rx, ui, an, tn, dm, mf, dv, kw, ac, sh, de, md, ip, vo, pg, sd, jn, pb, yr., ar, bs, bt, cf., dp, ja, pa, so] (40999)
25 couple*.mp. [mp = ti, ab, ot, nm, hw, kf, px, rx, ui, an, tn, dm, mf, dv, kw, ac, sh, de, md, ip, vo, pg, sd, jn, pb, yr., ar, bs, bt, cf., dp, ja, pa, so] (441164)
26 fathers*.mp. [mp = ti, ab, ot, nm, hw, kf, px, rx, ui, an, tn, dm, mf, dv, kw, ac, sh, de, md, ip, vo, pg, sd, jn, pb, yr., ar, bs, bt, cf., dp, ja, pa, so] (28088)
27 men*.mp. [mp = ti, ab, ot, nm, hw, kf, px, rx, ui, an, tn, dm, mf, dv, kw, ac, sh, de, md, ip, vo, pg, sd, jn, pb, yr., ar, bs, bt, cf., dp, ja, pa, so] (1998719)
28 sexual partner*.mp. [mp = ti, ab, ot, nm, hw, kf, px, rx, ui, an, tn, dm, mf, dv, kw, ac, sh, de, md, ip, vo, pg, sd, jn, pb, yr., ar, bs, bt, cf., dp, ja, pa, so] (23948)
29 prevention*.mp. [mp = ti, ab, ot, nm, hw, kf, px, rx, ui, an, tn, dm, mf, dv, kw, ac, sh, de, md, ip, vo, pg, sd, jn, pb, yr., ar, bs, bt, cf., dp, ja, pa, so] (806126)
30 reduc*.mp. [mp = ti, ab, ot, nm, hw, kf, px, rx, ui, an, tn, dm, mf, dv, kw, ac, sh, de, md, ip, vo, pg, sd, jn, pb, yr., ar, bs, bt, cf., dp, ja, pa, so] (4524890)
31 educat*.mp. [mp = ti, ab, ot, nm, hw, kf, px, rx, ui, an, tn, dm, mf, dv, kw, ac, sh, de, md, ip, vo, pg, sd, jn, pb, yr., ar, bs, bt, cf., dp, ja, pa, so] (1172156)
32 (awareness or health promotion or safe sex or condom* or efficacy or efficiency or behav* or test* or notif* or contact tracing* or prophylaxis* or counsel*).mp. [mp = ti, ab, ot, nm, hw, kf, px, rx, ui, an, tn, dm, mf, dv, kw, ac, sh, de, md, ip, vo, pg, sd, jn, pb, yr., ar, bs, bt, cf., dp, ja, pa, so] (8218275)
33 1 or 2 or 3 or 4 or 5 or 6 or 7 or 8 or 9 or 10 or 11 or 12 (466339)
34 13 or 14 or 15 or 16 or 17 or 18 or 19 or 20 or 21 (21121)
35 22 or 23 or 25 or 26 or 27 or 28 (2473213)
36 29 or 30 or 31 or 32 (12143165)
37 33 and 34 and 35 and 36 (1335)
38 remove duplicates from 37 (1048)

* = is a search tool used in narrowing the search related to a particular term

The outputs of the search were exported to Mendeley desktop 1.16.1 and duplicates were removed. After removal of duplicates in Mendeley, the titles and abstracts of the studies were screened independently by NFT and JAA. The full texts were obtained from the screened abstracts after inclusion and exclusion criteria were applied. Authors of articles were contacted for further information on any publication.

### Data collection process and data items

A data extraction spreadsheet was developed in excel version 2013. The data extraction sheet captured the following variables: the country of study which reflected the sub region of study; the sub region of study was divided according the four sub regions in SSA (East Africa, West Africa, Central and Southern Africa); the study design which could either by cohort studies or randomized controlled trials; the study population which captured the sample size; the study setting (urban, rural or both); the degree of male involvement; and the approaches/intervention used for PMTCT improvement.

The data spreadsheet also included information on the authorship and the odds ratios which is a measure of the impact of the approaches.

The outcome of interest was uptake of infant prophylaxis. Odds ratios were extracted from individual studies. Relative risks and proportions were converted to ORs. Two reviewers (NFT and JAA) independently extracted these data from the included studies. Any disagreement was settled by a third reviewer (LNA). The proportions and relative risks were converted into odds ratios, noting the effect size and the 95% confidence interval (CI). The characteristics of included studies were summarized in Table 2.

**Table 2:** Characteristics of included studies (variables and impact expressed in terms of odds ratios extracted from included studies)

Author	Study population	Study design	Approach used	Degree of male involvement	Unadjusted OR
Aliyu et al. 2016 [35]	364 HIV positive mothers and infants Nigeria	RCT	Complex community interventions	Antenatal clinic(ANC) attendance	3.04 (1.16–5.47)
Becker et al. 2010 [30]	81 HIV positive mothers and infants in Tanzania	RCT	Invitation letter	ANC attendance couple voluntary counselling and testing	4.31 (3.46–5.37)
Byamugisha et al. 2010 [36]	1713 HIV positive mothers and infants in Uganda	Cohort	Complex community interventions	Couple voluntary counselling and testing	1.35 (1.08–1.68)
Conkling et al. 2010 [29]	185 HIV positive mothers for each centre in Zambia	Cohort	Invitation letter	Couple voluntary counselling and testing	1.08 (0.73–1.57)
Conkling et al. 2010 [29]	185 HIV positive mothers a for each centre in Rwanda	Cohort	Invitation letter	Couple voluntary counselling and testing	1.21 (0.66–2.23)
Farquhar et al. 2004 [33]	217 HIV positive women and their infants in Kenya	Cohort	Enhanced psychosocial intervention	ANC attendance couple voluntary counselling and testing	3.4 (1.3–9.0)
Kalembo et al. 2013 [31]	476 HIV positive women and their infants in Malawi	Cohort	Verbal encouragement	HIV counselling and testing, HIV status disclosure and support to adhere to protocols	1.4 (0.5–3.8)
Peltzer et al. 2010 [32]	745 HIV positive mothers and infants in South Africa	Cohort	Verbal encouragement	ANC attendance	2.95 (2.03–4.3)
Weiss et al. 2014 [34]	25 HIV positive mothers and infants in South Africa	RCT	Enhanced psychosocial intervention	ANC attendance couple voluntary counselling and testing	4.0 (0.36–44.7)

*ANC* Antenatal clinic, *RCT* Randomized controlled trial, *HIV* Human Immunodeficiency Virus

### Synthesis of results and data analysis

The studies that were relevant after inclusion and exclusion criteria were applied were used in the synthesis. Studies with data on impact of male involvement on the uptake of infant prophylaxis were considered for a meta-analysis that was performed using statistical software Stata version 11.0. In this review the studies included varied significantly in terms of approaches and outcomes which suggests that the true effect sizes measured could also differ. This disparities could very likely introduce high heterogeneity. Therefore, the random effect model was used to pool the evidence from the studies.

Heterogeneity was assessed using the I squared statistic generated. Heterogeneity refers to the variation between the included studies and it was assessed as follows: if the I^2^ = 25%–49% we considered a “low” heterogeneity, if the I^2^ = 50%–74% we considered a “moderate” heterogeneity and if the I^2^ ≥ 75% we considered a “high” heterogeneity [26]. Meta-regression was used in exploring the reasons for heterogeneity between studies. Variables included in the regression were: the sub region of the study (grouped as Southern Africa, East Africa and West Africa); the study setting (urban, rural or mixed); the sample size. There is no standard definition of male involvement in the literature. To measure male involvement in a way that can encompass the practical assistance given by men to women and the various ways in which the men may overcome the gender norms in providing support to women is very challenging. In this study we incorporated several definitions of male involvement such as antenatal care attendance, couple HIV counselling and testing into a composite score or index. This composite score was used as a continuous variable in metaregression analysis.

### Quality assessment of studies

The Newcastle Ottawa scale was used in assessing the quality of non-randomized studies [27]. This scale captured 8 core elements divided into 3 broad elements related to the study quality. The first element was to determine the representativeness of the exposed cohort. The second element was to determine if the study controlled for other variables. The Third element was to determine if there was bias in the measurement of the outcome. A score of less than 4 was considered to be of low quality; a score of 4–5 was considered moderate quality and a score of greater than 6 was considered high quality.

The Jadad scale was used in scoring the methodological study of randomized studies (RCT) [28]. The scale has three components that include random sequence generation (randomization), allocation and blinding, and description of withdrawals/drop outs. The maximum score was 5. Studies were classified as having ‘high risk of bias’ if the Jadad score was 0 or 1, ‘moderate risk bias’ if the score was 2 or 3, and ‘low risk of bias’ if the score was 4 or 5.

## Results

**Fig. 1:**
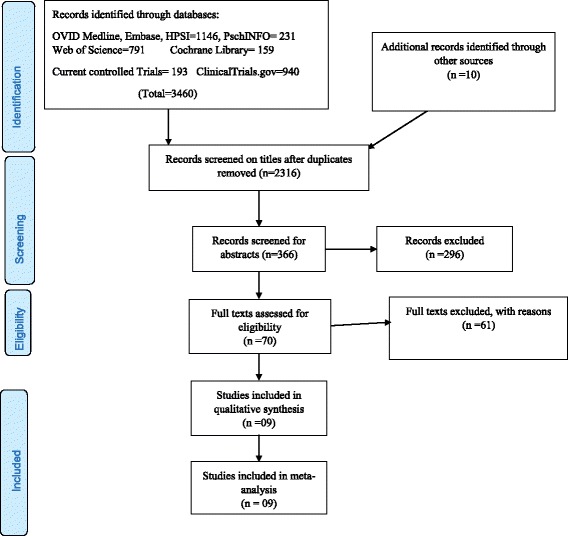
PRISMA flow diagram showing databases searched, screening and inclusion of studies

The electronic search on Ovid Medline, Ovid Embase, Ovid Health and Psychosocial instruments, Web of Science, Cochrane library, ClinicalTrial.gov, Current controlled trials returned 3460 results and after removal of duplicates this reduced to 2316 results. The study selection process is shown on the PRISMA flow diagram in Fig. 1.

Nine studies were finally included in the systematic review and meta-analysis. Of these 09 studies, three (03) were randomized trials while six (06) were cohort studies.

### Characteristics of included studies

Table 2 shows the characteristics of included studies. The study population of participants from the randomized controlled trials included a total of 470 HIV positive mothers and their infants. The cohort studies included a total of 3521 HIV positive mothers and their infants.

The sample size of the studies varied from 25 to 1713. Four out of nine studies were conducted in southern African countries such as South Africa (02 studies in 2009 and 2014), Malawi (01 study in 2013) and Zambia (01 study in 2010). In these southern African countries, the study conducted in South Africa in 2014 and the study in Malawi were conducted in a rural setting. Four out of the nine studies were conducted in east African countries such as Tanzania (01 study in 2009), Kenya (01 study in 2004), Uganda (01 study in 2010) and Rwanda (01 study in 2010). All these east African studies were conducted in urban settings. Only one study was conducted in the west African country of Nigeria in 2016. This study was conducted in the rural setting of northern Nigeria. The definitions of male partner involvement used in the studies include: antenatal care attendance, couple voluntary counselling and testing, HIV status disclosure.

In three of the included studies (one RCTs and two cohort), the investigators used invitation letters in order to improve the involvement of male partners in PMTCT [29, 30]. The two main types of invitation letters used were the ‘official invitation letter’ and ‘unofficial invitation letter’. The letters were considered official if they were signed by the head of the health facilities. However, even though the official letters were signed by the heads of the health facilities, the investigators made no effort in confirming if the letters were actually handed over to the male partners.

In two of the studies (all cohort studies) HIV positive mothers were encouraged verbally through counselling to bring their partners for counselling to the clinic [31, 32]. The two authors Kalembo et al. and Peltzer et al., gave no description of the verbal message given to the women or the personelle responsible to pass across the message [31, 32].

In two of the studies (one RCT and one cohort), the authors used psychological interventions that can be considered to be enhanced because they were conducted by trained personnel [33, 34]. Trained HIV positive personnel were used by Farquhar and colleagues while Weiss et al. used the Partnerplus intervention in which trained HIV facilitators were gender-matched to conduct cognitive and behavioural skill training. Weekly sessions were conducted for 90–120 min addressing issues of HIV counselling and behaviour.

In two of the studies (one RCT and one cohort), the investigators used complex community interventions in which several approaches to involve male partners were coupled with other changes in health care delivery to improve on maternal and child care within the community [35, 36]. Aliyu et al. used male champions who were considered to be role models in the community [35] while Byamugisha et al. focused only on the family with no public event organized in the community [36]. The large sample size of the studies that used complex community interventions meant they had high statistical power.

### Quality assessment

**Fig. 2:**
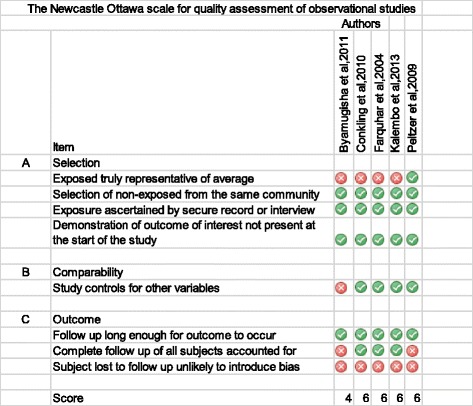
Quality assessment of observational studies included using the Newcastle Ottawa Scale

**Fig. 3:**
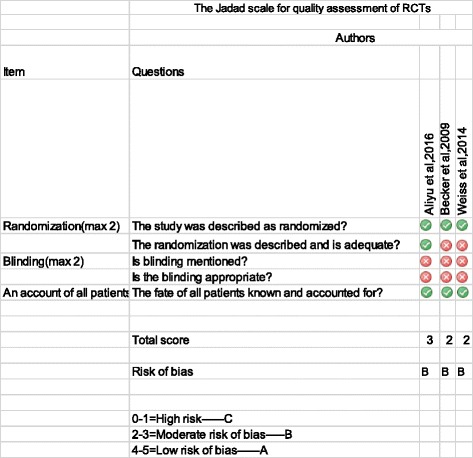
Quality assessment of randomized controlled trials included using the Jadad scale

Using the Jadad scale, the randomized trials had a moderate risk of bias while the observational studies had from moderate to low risk of bias using the Newcastle Ottawa Scale. The Figs. 2 and 3 show the results of the quality assessment.

### Results of meta-analysis and meta-regression

**Fig. 4:**
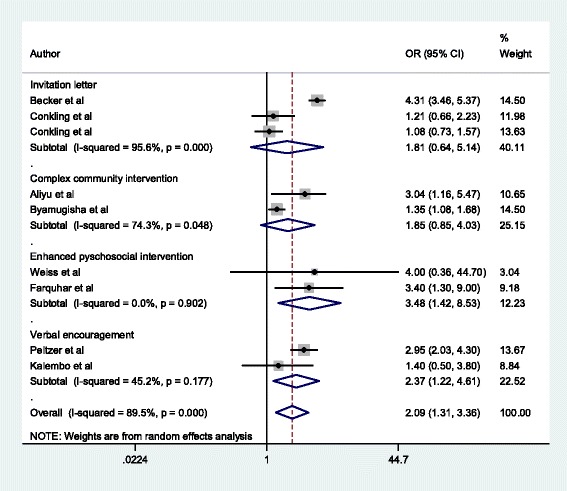
Forest plot showing the pooled effect sizes of each category of male involvement approach used in the included studies

Figure 4 shows the overall forest plot of the unadjusted odds ratios for the studies that reported the impact of male involvement on the uptake of infant prophylaxis. The pooled estimate of the odds ratios given by the blue diamond at the bottom is 2.09(95% CI: 1.31–3.36). From the same figure, the unadjusted odds ratios for enhanced psychosocial interventions (02 studies pooled), complex community interventions (02 studies pooled), verbal encouragement (02 studies pooled) and invitation letters (03 pooled studies) were 3.48(95% CI: 1.42 to 8.53), 1.85(95%CI: 0.85 to 4.03), 2.37(95%CI: 1.22 to 4.61) and 1.81(95%CI: 0.64 to 5.14) respectively. The I-squared =89.5%, *p* < 0.001). The sample size could explain the heterogeneity on meta-regression. The composite score that was used in capturing the definition/degree of male involvement could not also explain the heterogeneity on metaregression.

**Fig. 5:**
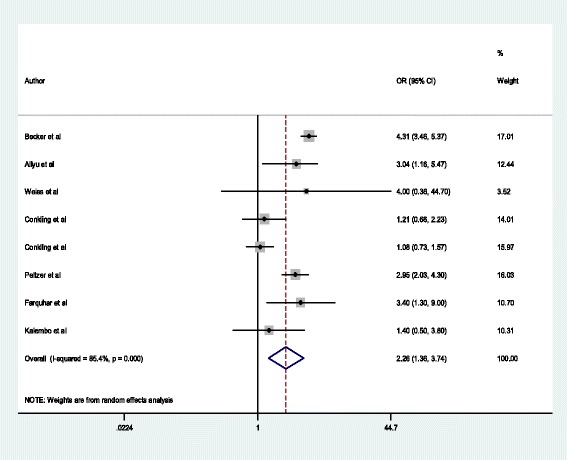
Forest plot showing results of sensitivity analysis when single large sample study was removed

When a sensitivity analysis was carried out by removing the study with the large sample size compared to the others, the pooled effect size only slightly increased to 2.26(95% CI: 1.36–3.74) and the heterogeneity was still high at 85.4%, p < 0.001 (Fig. 5).

**Fig. 6:**
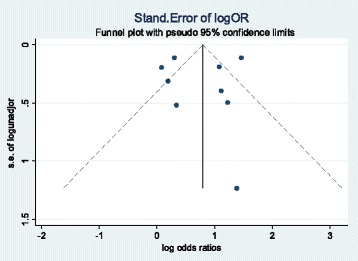
Funnel plot for studies reporting impact of male partner involvement approaches on the uptake of infant prophylaxis

The funnel plot in Fig. 6 shows an even distribution of point along the central axis. This shows there was no evidence for publication bias.

## Discussion

A meta-analysis of 09 odds ratios from 09 studies showed a statistically significant increase in the uptake of infant antiretroviral prophylaxis with male partner involvement. This increase was associated with enhanced psychosocial intervention and verbal encouragement. Invitation letters and complex community interventions had no effect on the uptake of infant antiretroviral prophylaxis. These findings are similar to previous studies carried out in the area of male partner involvement in PMTCT. In a narrative review, Auvinen et al. identified 3 main strategies to involve male partners in PMTCT which include: strategies that focus on the resources of the couples; strategies that focus on the development of health care; and community strategies [37]. In our study we also identified complex community strategies that encompass all the 3 strategies identified by Auvinen et al. However, we identified 3 additional approaches not mentioned in the Auvinen et al. study which include: invitation letters, verbal encouragement and enhanced psychosocial interventions. It should be noted that the study by Auvinen et al. was a narrative review with no systematic search strategy, no multiple database search, and no independent assessment of the quality of included studies. These limitations may explain why the current systematic review identified additional studies with other relevant approaches. In addition, the narrative review could not provide data on the impact of male involvement on the uptake of PMTCT services.

Yargawa and colleagues in a recent systematic review suggested that male involvement has a positive impact on maternal outcomes such as postpartum depression, utilization of hospital services and postnatal care [38]. Despite a robust search strategy and meta-analysis carried out, the study did not include essential PMTCT services related to the mother-infant dyad such as infant antiretroviral prophylaxis. Brusamento et al. conducted a systematic review focused on the impact of male involvement on the uptake of PMTCT services [19]. Even though Brusamento et al. thoroughly searched multiple databases with a comprehensive and systematic search strategy, independently assessed the quality of studies, they ended up with a single unblinded randomized controlled trial that met the inclusion criteria. The search strategy was biased towards identifying only randomized trials which could explain why the authors missed out relevant observational studies that could have improved on the evidence synthesis with possible meta-analysis. From this single study retrieved by the authors, the findings suggested that promoting male partner involvement in PMTCT through the use of invitation letters had no effect on the uptake of services such as infant prophylaxis. This finding by Brusamento et al. is in agreement with our finding that invitation letters had no impact on infant antiretroviral prophylaxis. However, unlike the Brusamento et al. study, our study suggests that approaches such as enhanced psychosocial intervention and verbal encouragement have a positive impact on the uptake of infant antiretroviral prophylaxis.

The use of invitation letters had important caveats which could explain why the letters had no impact on the uptake of infant antiretroviral prophylaxis. Firstly, there was the lack of confirmation by the authors if the women actually gave the letters to their male partners. This could have partially accounted for the low response rate observed in the studies leading to selection bias since the women selected likely did not share the content of the letter with their male partners. Secondly, the authors did not explore the educational level of male and female patients and their ability to read and understand the content of the written letters. They also did not explore the cultural factors that could affect the success of the letters. For example, the authors could have stated if the letters were written in the language that was well understood by the participants.

With respect to the use of complex community interventions, it was not vividly clear why this supposedly integrated and comprehensive approach had no impact on the uptake of infant prophylaxis. There are several strengths of complex community intervention that suggest they should have a positive impact on the uptake of infant prophylaxis. Firstly, there was a higher response rate which was likely due to more engagement [39]. By involving communities the authors went closer to the male partner than in other approaches mentioned so far. This could reduce the chances of measurement errors observed in other approaches that relied in report from the pregnant women. Secondly, using several approaches within the community meant the authors were likely to have explored diverse sociocultural context of these communities which could have accounted for the relatively higher response rate as compared with other categories of approaches used [40]. However, with just two studies pooled together in our study related to community interventions, our findings are likely conservative and hence more studies will be needed in the future that have used complex community interventions in order to confirm or refute our finding.

Our findings ultimately point towards the supremacy of integrating psychosocial components in male involvement interventions. Enhanced psychosocial interventions used either peer HIV counsellors with well-equipped communications skills or the PartnerPlus interventions in which trained HIV facilitators were gender-matched to conduct cognitive and behavioural skill training [34] Weekly sessions were conducted for 90–120 min addressing issues of HIV counselling and behaviour. The use of these behavioural techniques could be seen as advantageous because they may not only improve on male partner involvement in counselling and testing for HIV but also impact on their subsequent lifelong behavioural adaptations that could have long-term benefits for the family as a whole [41]. The use of trained facilitators and frequent weekly sessions may have been responsible for the high response rate of 87% observed which meant low selection bias. Even when non-specialist health workers are used, these psychosocial interventions have been shown to be very effective in tackling perinatal depression in view of improving maternal and child health [42]. Our study provides more evidence on their relevance in the domain of male involvement in PMTCT.

This systematic review and meta-analysis had several strengths and some limitations. The search was comprehensive because several databases and grey literature were searched and authors were contacted for any unpublished studies. There was also independent search and screening of articles by 2 reviewers which reduced bias. Since the search ended in March 2016, new articles published between this time and the submission of the manuscript could affect the findings. However, a final search was conducted on 31 December 2016 and no new articles relevant to our outcome of interest were retrieved. Unlike other systematic reviews on male partner involvement, the quality of studies included in our review was critically assessed. This assessment showed that the RCTs were prone to observer bias since there was no blinding. Despite this weakness, blinding was not feasible because a knowledge of the couples was essential in guiding the interventions. In the studies included for the systematic review, none of the observational studies adjusted for confounders. The findings of the study were likely strongly affected by confounding. However, for randomized trials, the randomization reduced the selection bias significantly while the confounding was reduces by matching. Another limitation worth mentioning is the lack of generalizability resulting from the small number of studies and samples sizes as well as very few articles from west African countries. In addition, the heterogeneity was very high among studies suggesting that the studies were not similar and may be could not be combined in a meta-analysis. Despite this high heterogeneity, it should be noted that this is the first meta-analysis to explore the impact of male partner involvement on the uptake of a PMTCT service such as infant antiretroviral prophylaxis. Our study therefore provides suggestions for more studies in future in order to improve on the quality of evidence in this very important aspect of HIV research in sub Saharan Africa.

## Conclusion

Our study shows that the approaches used in improving male partner involvement that targeted the uptake of infant prophylaxis are enhanced psychosocial intervention, verbal encouragement, invitation letters and community intervention. Our findings suggest that there is stronger evidence that enhanced psychosocial interventions and verbal encouragement increased the uptake of infant prophylaxis. The heterogeneity among studies was high and was unexplained. The high heterogeneity and small sample size suggests more studies are needed to draw a definite inference from the meta-analysis.

### Recommendations for future research

Few randomized trials have been carried out to investigate the impact of male partner involvement on the uptake of infant prophylaxis. More randomized trials are needed to add to the strength evidence available. More studies with larger sample sizes and which are conducted using similar methodology are needed in future. Finally, an economic evaluation is needed to adequately inform policy.

## Abbreviations

HIV: Human immunodeficiency virus
PMTCT: Prevention of mother to child transmission
PRISMA: Preferred reporting items for systematic reviews and meta-analyses
PROSPERO: International prospective register of systematic reviews
HPSI: Psychosocial instruments
PsycINFO: Psychological information
RCT: Randomized controlled trial
SSA: Sub Saharan Africa
UNAIDS: Joint United Nations Programme on HIV/AIDS
